# Complete response in a patient with liver metastases from breast cancer employing hepatic arterial infusion 5-fluorouracil based chemotherapy plus systemic nab-paclitaxel

**DOI:** 10.18632/oncotarget.23793

**Published:** 2017-12-31

**Authors:** Girolamo Ranieri, Ilaria Marech, Mariangela Porcelli, Francesco Giotta, Gennaro Palmiotti, Giuseppe Laricchia, Vito Fazio, Cosmo Damiano Gadaleta

**Affiliations:** ^1^ Interventional and Medical Oncology Unit, National Cancer Research Center, Istituto Tumori Giovanni Paolo II, Viale Orazio Flacco, Bari, Italy

**Keywords:** hepatic intra-arterial chemotherapy, nab-paclitaxel, liver metastases, breast cancer

## Abstract

About half of patients with metastatic breast cancer (mBC) have unresectable liver metastases (LMs) or liver-predominant disease (LPD). Unfortunately systemic chemotherapy has limited tumor response due to LMs are supplied by hepatic artery. Hepatic intra-arterial (HAI) have antitumor activity in pretreated patients with LMs. Here we report the case of a 55-year-old woman affected by BCLPD and heavily pretreated. LMs responded to treatment based on HAI with 5-fluorouracil and nab-paclitaxel systemic chemotherapy, and they completely disappeared on a CT-scan. We conclude that this combination chemotherapy is safe and may be very useful for the treatment of patients with BCLPD. Therefore, this combination should be evaluated in a large study.

## INTRODUCTION

Liver metastases (LMs) develop in approximately half of the women with metastatic breast cancer (mBC) and are typically associated with metastases at other sites, indicating advanced disease and poor prognosis [1]. Generally, systemic chemotherapy remains the first treatment option for patients with mBC, but patients with LMs have a poor tumor response In fact, LMs derive most of their blood supply from the hepatic artery [2].

Hepatic intra-arterial (HAI) chemotherapy has antitumor activity in selected heavily pretreated patients with LMs or liver-predominant disease (LPD). Therefore, the advantage of administering chemotherapy *via* HAI results in higher local concentrations than those achieved by intravenous (iv) infusion [3]. In particular, HAI therapy with floxuridine and dexamethasone plus systemic chemotherapy has shown clinical benefit in patients with colorectal LMs, as adjuvant therapy after resection, as salvage therapy, and as conversion therapy for unresectable disease [4–8]. For what concern BC, there are some reports of good response rates (RR) of LMs from BC by HAI and systemic chemotherapy [9–22].

In patients with BCLPD, the importance to administer systemic chemotherapies to HAI chemotherapy may reduce the risk of systemic progression. Therefore, the association of HAI with systemic chemotherapy may achieve clinically relevant disease control in patients with BCLM or BCLPD. In our case report we have observed a liver complete response using HAI with 5-fluorouracil plus systemic nab-paclitaxel in a woman with BCLPD who have progressed despite multiple prior lines of therapy.

## CASE REPORT

A 55-year-old woman born in Brindisi (Puglia, Italy), without history of hereditary BC, underwent right radical mastectomy plus axillary lymph nodes dissection for BC in January 2012. Pathologic stage was pT3N3M1 (stage IV) grade 3 infiltrating ductal carcinoma of right breast with bone metastases. Biological characterization was: estrogen receptor (ER) positive (60%), progesterone receptor (PgR) and human epidermal growth factor receptor 2 (HER2) negative, Ki-67 proliferation index: 24%.

After surgery, first line chemotherapy with liposomal doxorubicin (60 mg/m^2^ on day 1) and cyclofosfamide (600 mg/m^2^ on day 1) triweekly for six months plus zoledronic acid (4 mg on day 1 every 4 weeks) was administered in Medical Oncology Unit in Brindisi. In July 2012 hormonal therapy with tamoxifen (20 mg/day) was started and the patient received palliative radiotherapy on the painful bone metastases (right femoral neck). In November 2013, it has been found by computed tomography (CT) scan bone disease progression, therefore second line chemotherapy (gemcitabine 1000 mg/m^2^ on days 1 and 8 plus docetaxel 75 mg/m^2^ on day 1 triweekly) plus hormonal therapy (letrozole 2,5 mg daily) was given. In addition, the patient received palliative radiotherapy to the spine (C7-D2) and to left sacroiliac joint. In July 2015 third line systemic therapy with exemestane (25 mg/day) plus everolimus (10 mg/day) was started due to the further bone disease progression.

**Figure 1:**
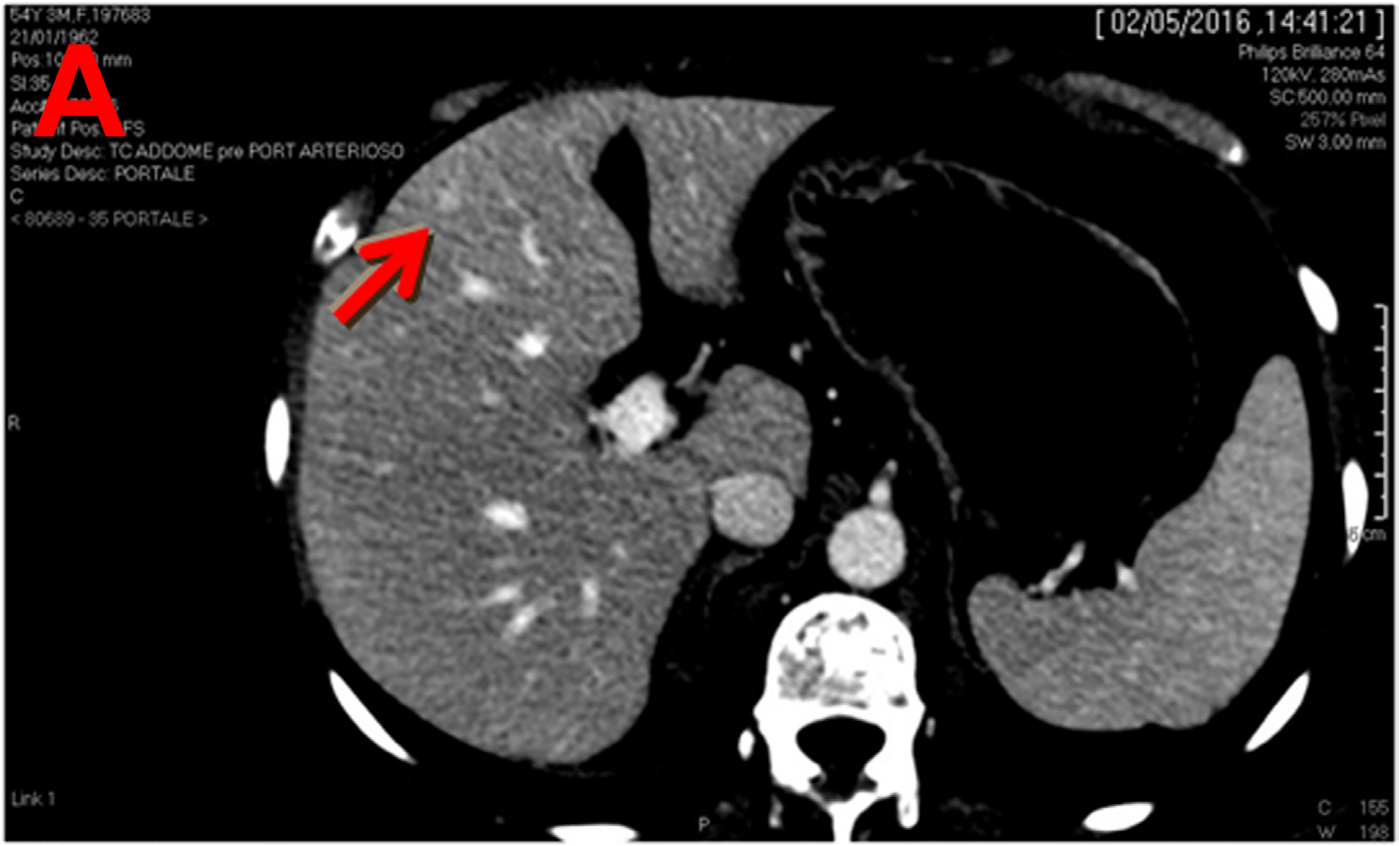

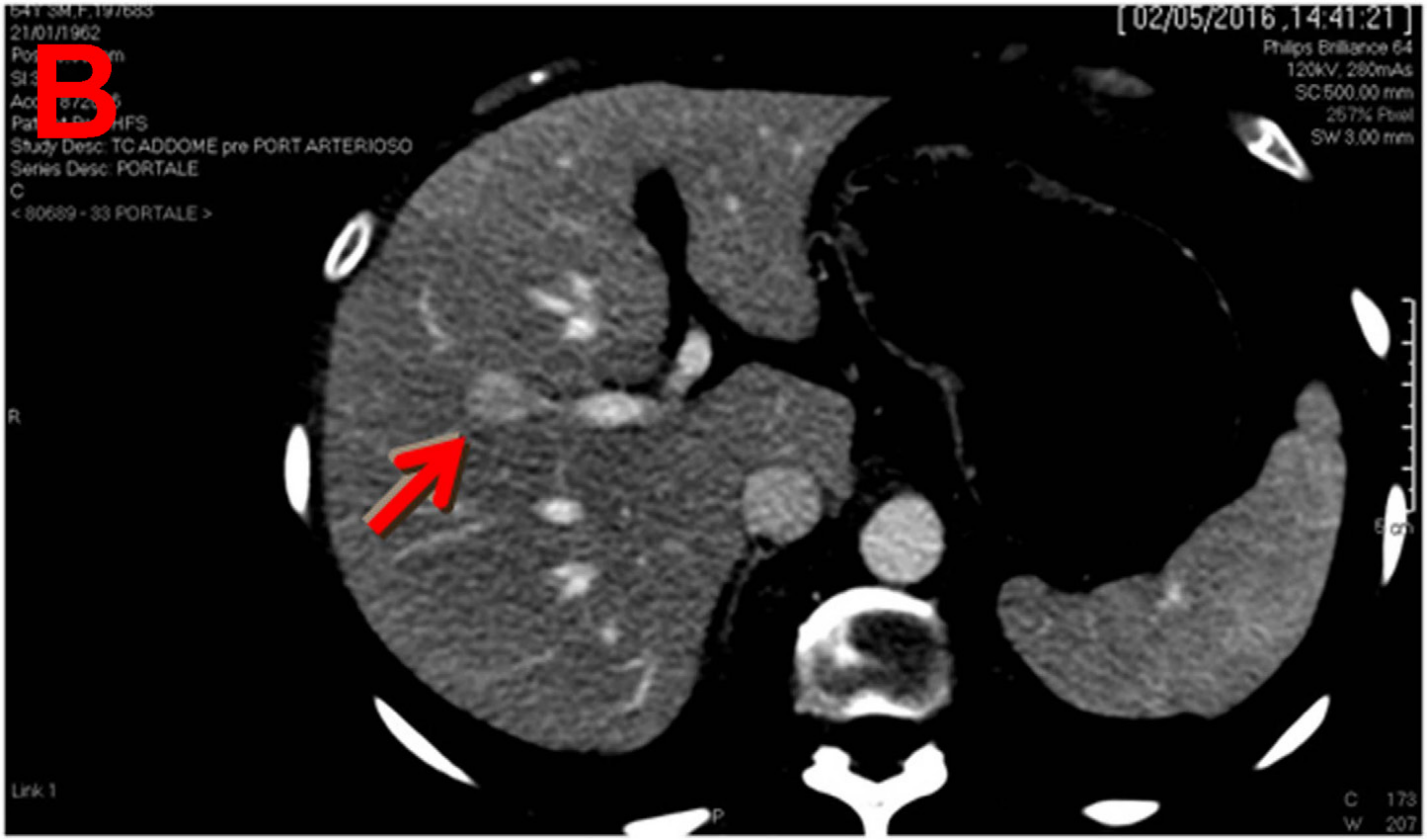

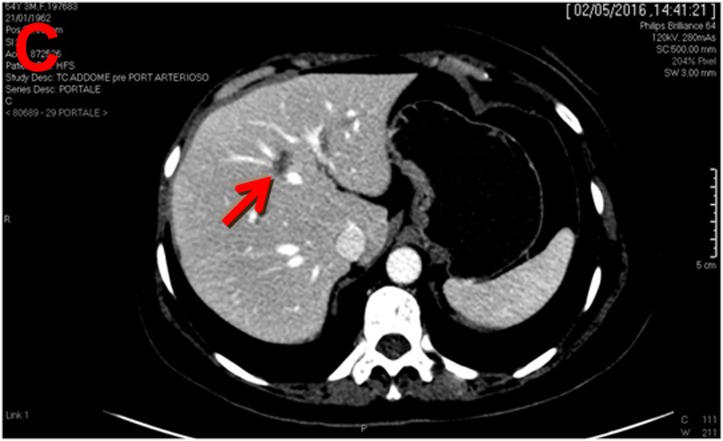
Computed tomography (CT) scan performed on April 2016 shows three liver lesions (LLs) in arterial phase **a.** The arrow indicates LL (diameter: 7 mm) with contrast enhancement suspected of metastasis at 4^th^ segment; **b.** The arrow indicates LL (diameter: 19 mm) with contrast enhancement suspected of metastasis at 5^th^ segment; **c.** The arrow indicates LL (diameter: 18 mm) without contrast enhancement, doubtful lesion for metastasis at 8^th^-4^th^ segment and in differential diagnosis with angioma.

In April 2016 CT scan showed two liver lesions (the first with 7 mm of diameter at 4^th^ segment - Figure 1a; the second with 19 mm of diameter at 5^th^ segment - Figure 1b) suspected of metastasis and a third lesion (with diameter of 18 mm at 8^th^-4^th^ segment) doubtful for metastasis and in differential diagnosis with angioma at 8^th^-4^th^ segment.

In May 2016 the patient underwent eco-guided agobiopsy of lesion at 8^th^-4^th^ segment in our Division (Interventional and Medical Oncology Unit, National Cancer Research Centre, Istituto Tumori “Giovanni Paolo II”, Bari, Italy). Histological exam confirmed metastases from BC. Fourth line of systemic chemotherapy with capecitabine (1000 mg/m^2^ twice daily, on days 1-14 triweekly) and oral vinorelbine (60 mg/m^2^ on days 1 and 8 of the first cycle and escalated to 80 mg/m^2^ at subsequent cycles) was administered. This chemotherapy was discontinued after 1 month due to unacceptable toxicity (gastrointestinal pain and hand-foot syndrome not responding to medical treatment).

**Figure 2:**
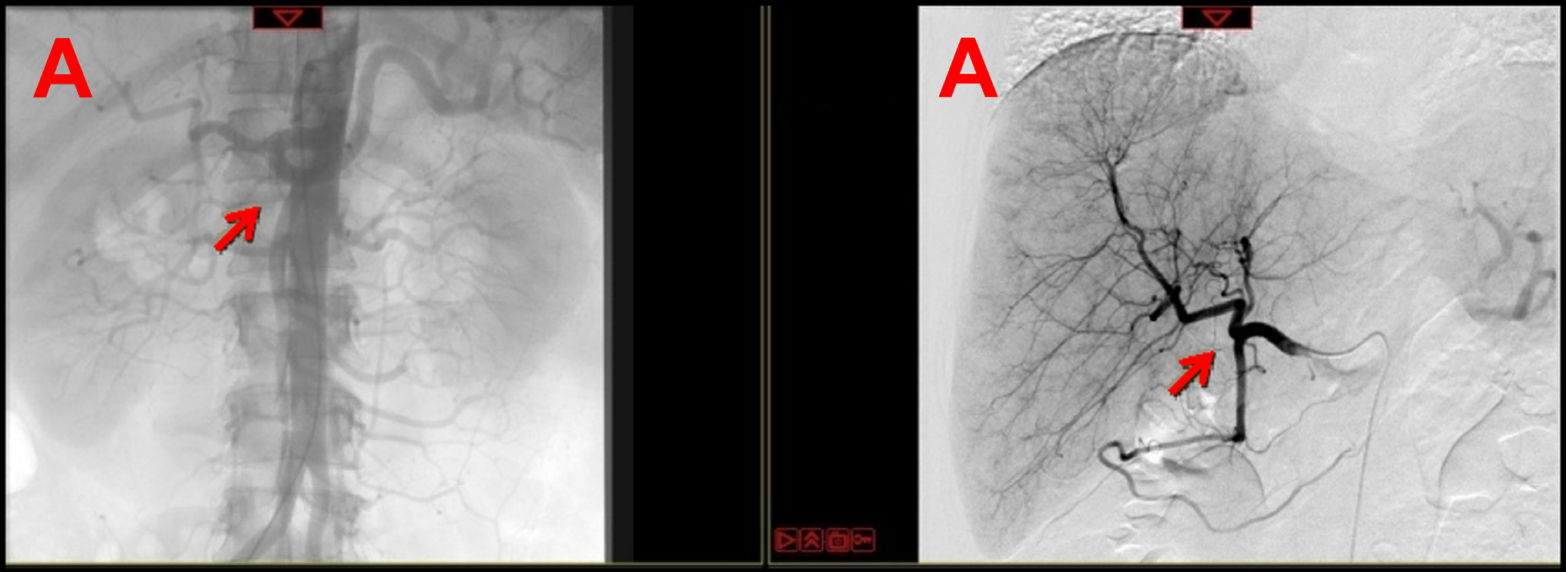

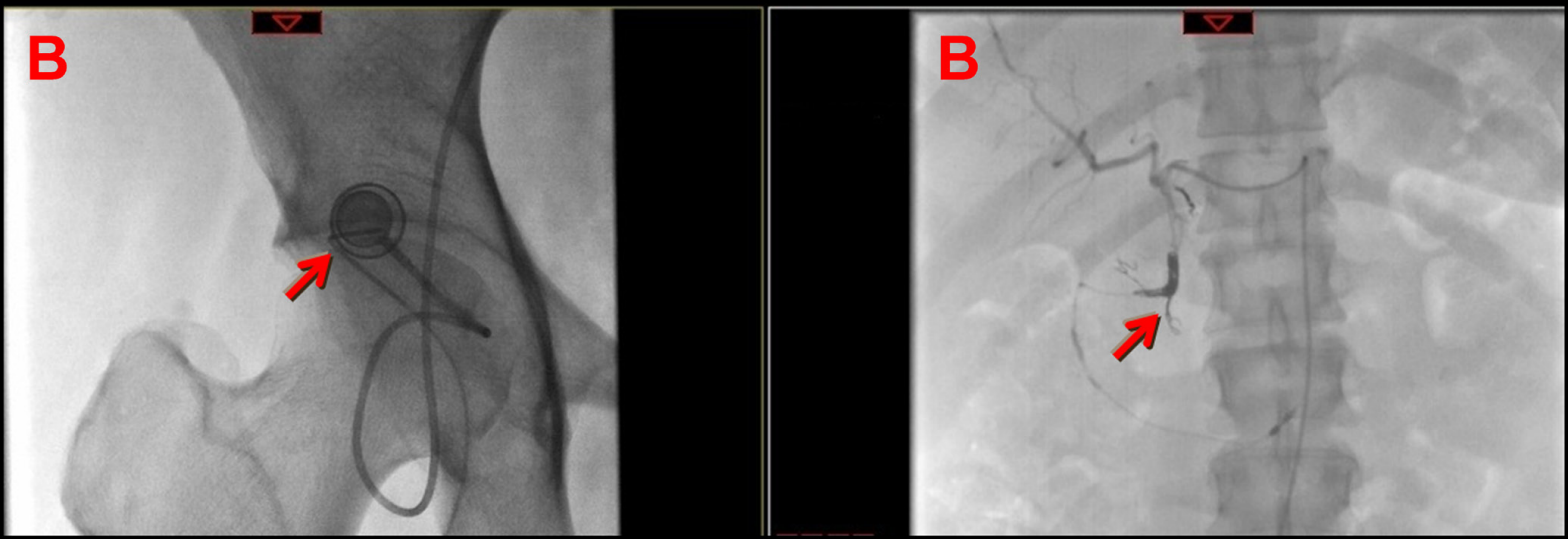
Arterial phase of contrast angiogram performed before (a) and after (b) the placement of the subcutaneous port and of the hepatic-artery catheter **a.** Arterial phase of contrast angiogram shows the celiac trunk (left arrow) and the preparation of the vascular bed (right arrow); **b.** Arterial phase of contrast angiogram shows the subcutaneous port with its connection system (left arrow) and the hepatic-artery catheter (right arrow).

In July 2016 the patient decided to continue treatments in our Division. Following collegial discussion between interventional radiologists and medical oncologists a subcutaneous pump was implanted after contrast angiogram (Figure 2) as described by Kemeny and Fata [4].

HAI of 5-fluorouracil (1200 mg/m^2^) dissolved in 100 ml of physiological saline was performed for 48 hours in continuous infusion using the installed reservoir by elettro-mechanic computered pump every two weeks. HAI was combined to folinic acid (100 mg/m^2^ daily) iv in two hours for 2 consecutive days every two weeks. In association with HAI she received iv nab-paclitaxel (260 mg/m^2^ on day 1) triweekly. Furthermore, iv zoledronic acid treatment was continued. During therapy, no serious side effects and only grade 1 abdominal pain were observed.

**Figure 3:**
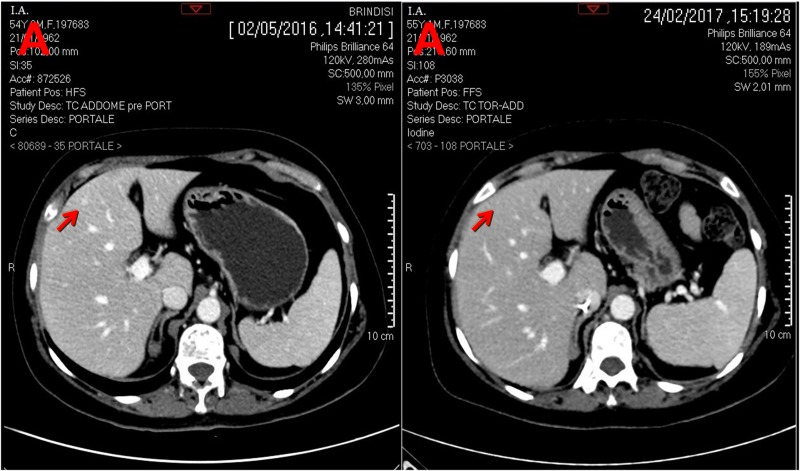

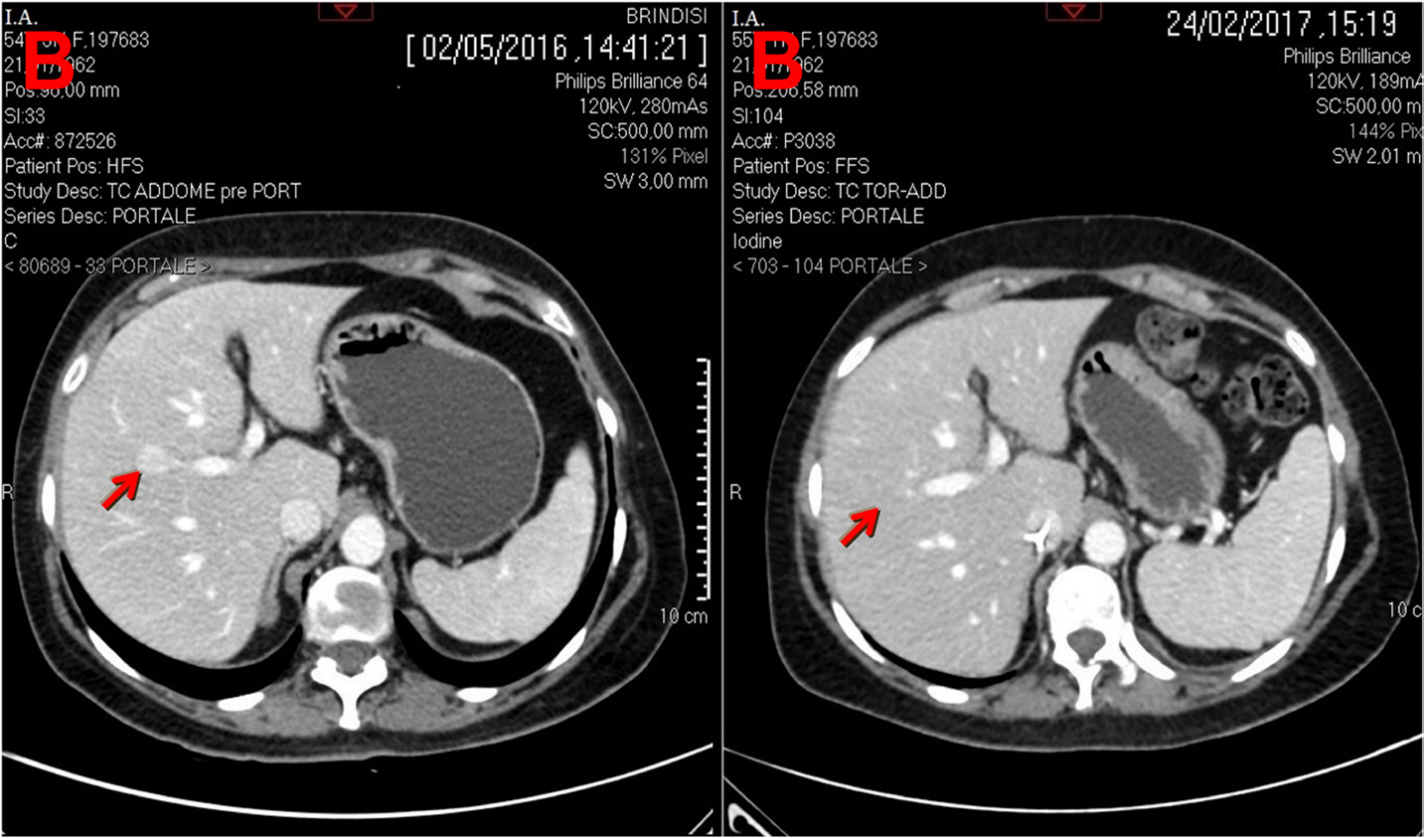

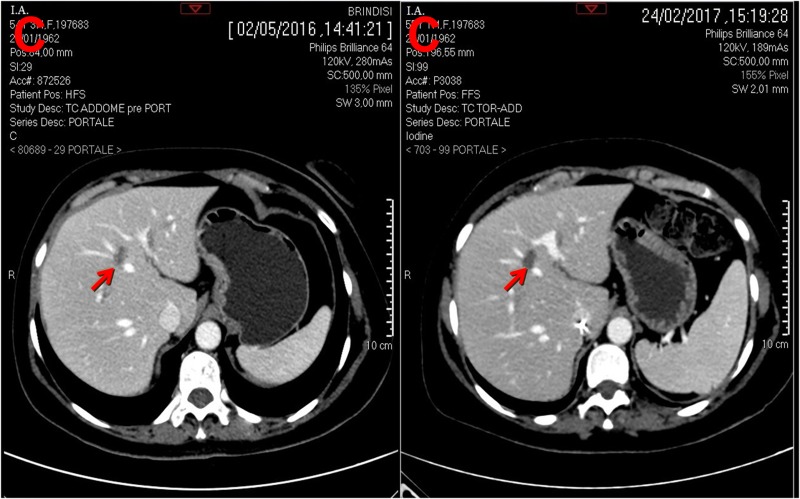
CT scan (portal phase) performed before and after hepatic artery infusion plus systemic chemotherapy **a.** Left arrow indicates LM at 4^th^ segment and right arrow indicates its complete disappearance at 4^th^ segment; **b.** Left arrow indicates LM at 5^th^ segment and right arrow indicates its complete disappearance at 5^th^ segment **c.** Left arrow indicates LM at 8^th^-4^th^ segment and right arrow indicates LM completely necrotic at 8^th^-4^th^ segment.

**Figure 4:**
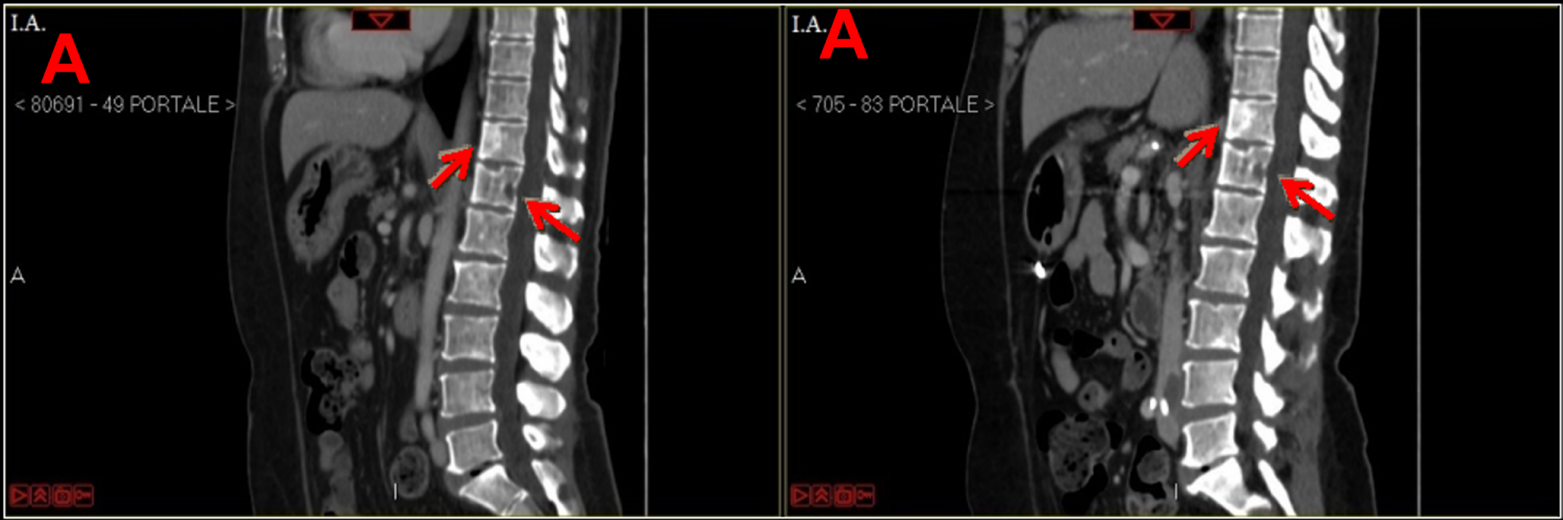

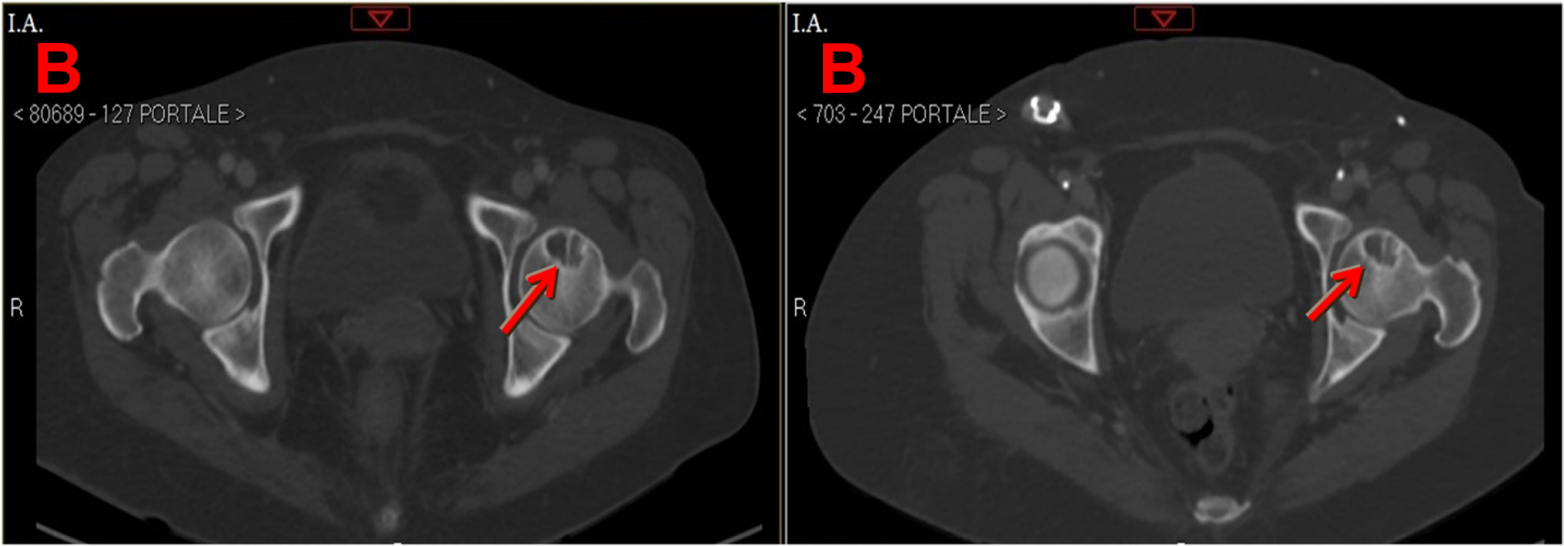
CT scan (portal phase) performed before and after hepatic artery infusion (HAI) plus systemic chemotherapy **a.** The upper left arrow and the lower left arrow indicate osteoblastic bone metastasis at D11 and osteolytic bone metastasis at D12, respectively before HAI plus systemic chemotherapy; the upper left arrow and the lower left arrow indicate osteoblastic bone metastasis at D11 osteolytic bone metastasis at D12, respectively, that seem unchanged after HAI plus systemic chemotherapy. **b.** The left arrow indicates osteolytic bone metastases at left femoral neck before HAI plus systemic chemotherapy; the right arrow indicates osteolytic bone metastases at left femoral neck that seem unchanged after HAI plus systemic chemotherapy.

After 16 cycles of HAI LMs responded to the treatment, and they completely disappeared on a CT scan (Figure 3a-3c). Bone disease was stable on the same CT scan (Figure 4a, 4b). In addition, elevated CA-15.3 in serum decreased to the normal range.

## DISCUSSION

We have reported the case of a 55-year-old woman with BCLPD inefficaciously pretreated with systemic chemotherapy from 2012. She came to our observation following visceral disease progression and after receiving multiple lines of treatment that included eleven systemic drugs (liposomal doxorubicin, cyclofosfamide, zoledronic acid, tamoxifen, gemcitabine, docetaxel, letrozole, everolimus, exemestane, capecitabine, vinorelbine). Today, after 16 cycles of HAI with 5-fluorouracil we have observed an apparent complete response on CT-scan associated to the normalization of tumor marker CA 15.3.

Patients with unresectable LMs or BCLPD are generally candidates for systemic chemotherapy that is the mainstay of treatment with a growing, but uncertain role for liver-directed therapies. Unfortunately systemic chemotherapy may prolong survival to a median of 12-18 months.

HAI chemotherapy has antitumor activity in patients with LMs from BC [20]. The most important rationale of HAI chemotherapy is that LMs are perfused almost exclusively *via* the hepatic artery, whereas normal hepatocytes derive most of their blood supply from the portal circulation [2]. Therefore, the advantage of administering chemotherapy *via* HAI results in higher local concentrations than those achieved by intravenous infusion [3]. In particular, 5-fluorouracil/floxuridine is a good drug for hepatic arterial chemotherapy because of the high total body clearance and high hepatic extraction rate [4]. For what concern clinical trials, there are some reports of good objective RR - ORR - (from 50-70%) of LMs from BC by HAI chemotherapy [9–21]. In pretreated mBC patients with LMs or BCLPD it has been evaluated various HAI chemotherapies (such as 5-fluorouracil/floxuridine, adriamycin, nabpaclitaxel, vinblastine, cisplatin/oxaliplatin, mytomicin C, irinotecan) alone [10, 14–17, 19, 21, 23] or associated with systemic chemotherapy [9, 11, 12, 22] or with hormonal therapy [9, 18]. Regarding HAI with 5-fluorouracile, several studies have evaluated HAI 5-fluorouracil chemotherapy based regimen with different dosage and schedules associated with various systemic drugs (such as taxane, anthracycline, capecitabine, gemcitabine, oxaliplatin) [9, 11, 12, 22] or monoclonal antibodies (bevacizumab, trastuzumab) [9, 11]. Interestingly, the JCOG study 9113 has analyzed safety and efficaciousness of HIA 5-fluorouracil chemotherapy based regimen observing an ORR of 63% and a median overall survival (mOS) of 25 months in patients with BCLPD [10]. Ang et al. have shown in pretreated patients with LMs who received HAI 5-fluorouracil chemotherapy based regimen plus several iv drugs a RR of 78% and a median OS of 17 months. Toxicity of HAI chemotherapy was manageable and mild, mainly involved bone marrow suppression and gastrointestinal tract symptoms. Catheter-related events were not dose dependent and the most frequent event was thrombosis chemotherapy [9–22].

In BCLPD, the importance to administer systemic drugs to HAI chemotherapy may reduce the risk of systemic progression. Therefore, the association of HAI with systemic chemotherapy may achieve clinically relevant disease control in patients with BCLM or BCLPD. We have chosen nab-paclitaxel as fifth line systemic chemotherapy that it is the current line treatment for BC after failure of combination chemotherapy for metastatic disease [24]. In the pivotal study, for patients who received nab-paclitaxel as second-line or further therapy beyond second-line, there was an ORR of 27% compared with an ORR of 13% for patients treated with paclitaxel (*p* = 0.006) [24]. Interestingly, it has been showed a significant increase of mOS in nab-paclitaxel arm (14 months) compared to the paclitaxel arm (12 months) only in patients who received second-line or greater therapy (HR: 0.73; *p* = 0.024) [24]. Considering the mORR (between 15-40%) of systemic chemotherapy and the mORR (from 50-70%) of HAI chemotherapy in mBC pretreated patients, the combination therapy (HAI plus systemic chemotherapy) may be represent a valid option to increase mORR and to improve mOS in patients with BCLM or BCLPD.

In particular, we conclude that HAI with 5-fluorouracil plus systemic nab-paclitaxel, is safe and may be very useful for the treatment of BCLPD, identifying a novel therapeutic schedule that could be intriguing to test in clinical trials.
